# Cell Proliferation in Cubozoan Jellyfish *Tripedalia cystophora* and *Alatina moseri*

**DOI:** 10.1371/journal.pone.0102628

**Published:** 2014-07-21

**Authors:** Daniela Gurska, Anders Garm

**Affiliations:** Marine Biological Section, Department of Biology, University of Copenhagen, Copenhagen, Denmark; UC Irvine, United States of America

## Abstract

Cubozoans (box jellyfish) undergo remarkable body reorganization throughout their life cycle when, first, they metamorphose from swimming larvae to sessile polyps, and second, through the metamorphosis from sessile polyps to free swimming medusae. In the latter they develop complex structures like the central nervous system (CNS) and visual organs. In the present study several aspects of cell proliferation at different stages of the life cycle of the box jellyfish *Tripedalia cystophora* and *Alatina moseri* have been examined through *in vivo* labeling of cells in the synthetic phase (S phase) of the cell cycle. Proliferation zones were found in metamorphosing polyps, as well as in juvenile medusae, where both the rhopalia and pedalia have enhanced rates of proliferation. The results also indicate a rather fast cell turnover in the rhopalia including the rhopalial nervous system (RNS). Moreover, *T. cystophora* showed diurnal pattern of cell proliferation in certain body parts of the medusa, with higher proliferation rates at nighttime. This is true for two areas in close connection with the CNS: the stalk base and the rhopalia.

## Introduction

Cell proliferation serves two purposes in all organisms: growth and maintenance/cell turnover. Both these functions are normally important throughout the life history of an animal but especially so during certain processes like metamorphosis where many new cell types are needed. Cnidarian medusae are the result of polyp metamorphosis, and this change is highly interesting since the animal changes from a sessile to a free living form. In this change a great expansion of the nervous and sensory systems is called for.

**Figure 1:**
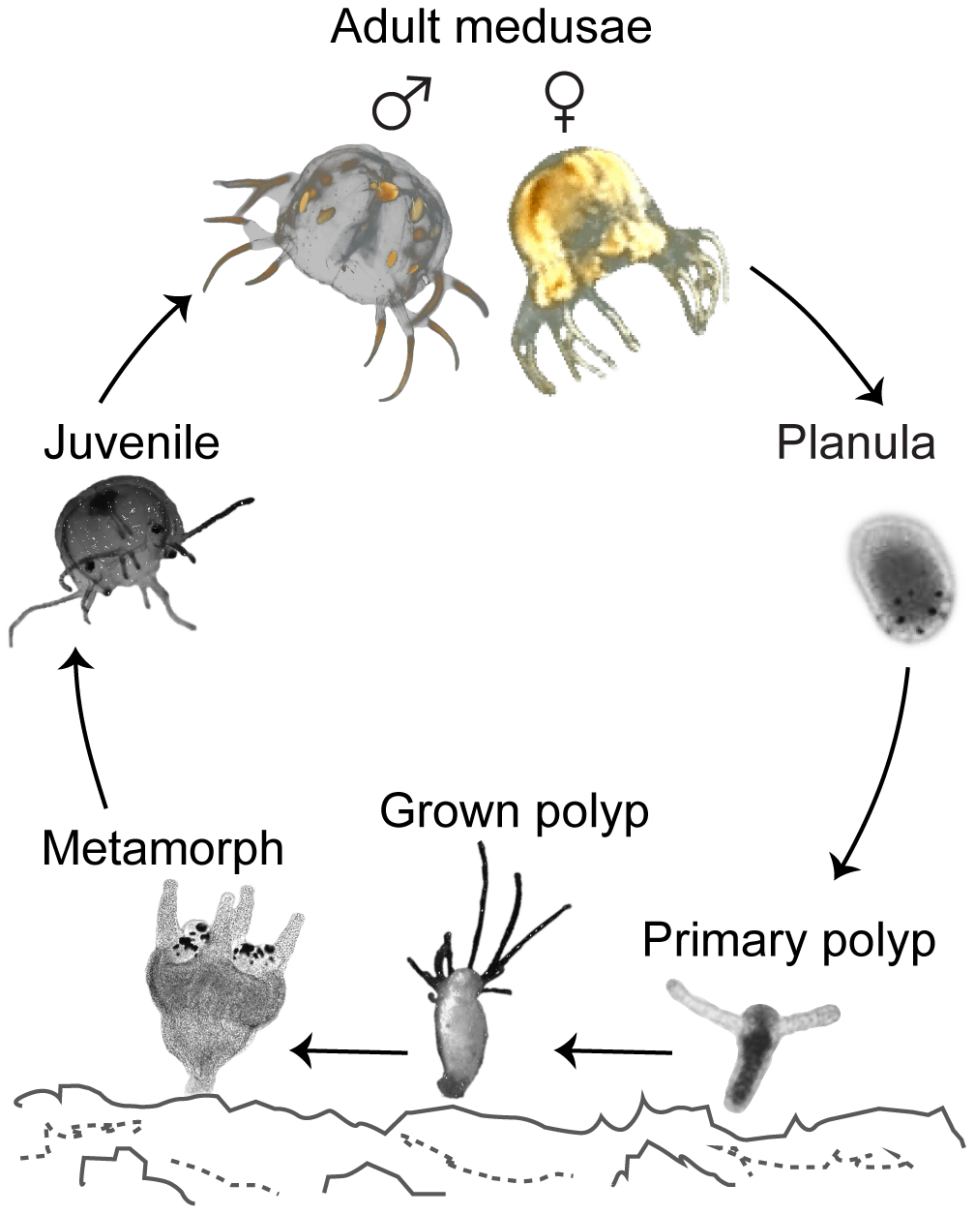
Life cycle of *Tripedalia cystophora*. Schematic diagram illustrating the life cycle of *T. cystophora*. The planula larva settles on the bottom and undergoes the first metamorphosis into a sessile primary polyp with two tentacles. The primary polyp grows into a fully grown polyp that usually possesses from 7 to 9 tentacles. At this stage much asexual reproduction takes place (not shown). Under optimal conditions the grown polyp undergoes the second metamorphosis and forms juvenile medusa with four primary tentacles. Sexual maturity of the medusae is reached in approximately three months. Fertilization is internal and the diagram shows a pregnant female with bell completely filled by larvae and an adult male with ripe spermatophores (orange spheres).

Cubozoans (Cnidaria) have a complex life cycle including planula larvae, sessile polyps and free swimming medusae (Figure 1). Among cnidarians only cubozoans undergo a complete metamorphosis from polyp to medusa in that the entire polyp turns into a single medusa [1], [2]. The cubozoan polyp has to undergo severe body reorganization and among other things it develops complex visual organs. The first sign of metamorphosis is the transformation of the circular oral pole into a quadrangular shape (Figure S1). The polyp tentacles then congregate at the four corners while the distal part of the tentacles degenerate and is reabsorbed [3]. Ultimately the basal part of the polyp tentacles, either singly or as a fused group, become the four eye carrying structures, called rhopalia, and in-between the rhopalia four medusa tentacles grow *de novo*. Once the external signs of metamorphosis appear in the Caribbean species *Tripedalia cystophora* and the conditions are optimal (water temperature 28°C) one polyp is completely converted into a single medusa in 4 to 5 days [3]. Here the new juvenile medusae have four primary tentacles, but during the first week a new small tentacle appears on each side of primary ones. Sexual maturity of the medusae is reached in 10–12 weeks.

Cubozoans also stand out from all other cnidarians by possessing a remarkable visual system. This visual system is distributed on the four rhopalia, which hang by flexible stalks near the rim of the box-shaped bell of the medusa. Altogether they have 24 eyes of four morphological types [4]–[8]. Each rhopalium carries a pair of pit eyes, a pair of slit eyes and two lens eyes: the upper lens eye (ULE) and the lower lens eye (LLE). It has been shown that the medusae use this unique visual system for optimizing feeding, avoiding obstacles and navigation in their habitats [9]–[11].

As previously described [12] each rhopalium also contains a major part of the central nervous system (CNS); the rhopalial nervous system (RNS). Most of the visual information is presumably processed by the approximately 1000 nerves forming the RNS, sometimes referred to as the box jellyfish brain [13], but little is known about the functionality of cnidarian CNSs. Most is known from hydrozoans [14], [15], and interestingly it has been indicated that in the hydrozoan *Hydra oligans*, there is a relatively fast cell turnover in the ring nerve, which here constitutes the CNS [16]. In the cubozoan rhopalia, in close connection with the RNS, three-four layers of cells (posterior cell sheet) are found and from their appearance in TEM they are assumed to be undifferentiated [17]. A possible function of the posterior cell sheet might be, therefore, to serve as a source of new nerve cells and also support a fast cell turnover in the rhopalium including in the RNS.

In this study we took the advantage of *in vivo* labeling of cells in the synthetic phase (S phase) of the cell cycle [18], to examine some morphological details of the metamorphosis from polyp to juvenile medusa of the cubozoans *T. cystophora* and *Alatina moseri*. We have also examined proliferation patterns in adult non-growing rhopalia to test the hypothesis, that the RNS has a cell turnover acquiring new cells originating from the posterior cell sheet. Finally, we examined a possible diurnal rhythm in the proliferation in *T. cystophora* hypothesized from the diurnal activity pattern described for the species which rest at night [19].

## Materials and Methods

### Cultures

The material used came from our cultures at University of Copenhagen. The cultures of *T. cystophora* originate partly from Werners cultures [3] and partly from pregnant females collected at La Parguera, Puerto Rico (no specific permissions required, no endangered or protected species were collected, GPS coordinates: 17°15′24.0′′N, 67°04′03.7′′W). The polyps are kept in 50 l tanks at 22°C in darkness and a salinity of 3.0 psu. The medusae of *T. cystophora* are raised in 250 l tanks at 28°C and a salinity of 3.0 psu where they reach adult size (bell diameter  = 9–10 mm) in about 10 weeks. The medusa tanks had a day:night cycle of 8:16 h with light between 0900 hr and 1700 hr. The cultures of *A. moseri* were established by mixing ripe eggs and sperm from medusae caught off the coast of Hawaii. The culture tanks are similar to those of *T. cystophora* except for having a salinity of 3.5 psu. All culture tanks are fed SELCO (INVE Technologies, Dendermonde, Belgium) enriched artemia daily.

### Labeling protocols

Proliferating cells were visualized by *in vivo* labeling using a thymidine analogue 5-ethynyl-2′-deoxyuridine (EdU) that is being incorporated into DNA instead of thymidine during the S phase of the cell cycle. Polyps and medusae of *T. cystophora* and *A. moseri* were incubated with 20 µM EdU (Click-iT EdU Kit, catalogue number C10424, Life Technologies Europe BV, Nærum, Denmark) for different lengths of time (see later). After EdU treatment the specimens were anesthetized with 4% MgCl_2_ in sea water and fixed with 4% paraformaldehyde in 0.1 M phosphate-buffered saline (PBS), pH = 7.3 for 4 h at room temperature or overnight at 4°C. This procedure was followed by 3 washes (15 min each) with 0.1% NaN_3_ in 0.1 M PBS, pH = 7.3. Until further processing the samples were stored in 0.1% NaN_3_ at 4°C. After storage they were rinsed in 0.1 M PBS, pH = 7.3 for 6 h followed by overnight incubation in blocking and permeabilization solution (saponin-based permeabilization and wash reagent with 1% NSS provided by the manufacturer) at 4°C. The following day the samples were incubated in the reaction cocktail provided by the manufacturer (2.5 µl Alexa 488, 10 µl CuSO_4_, 50 µl Reaction buffer additive, 438 µl 0.1 M PBS, pH = 7.3) for 24 h at 4°C in the dark. The incubation in the reaction cocktail was followed by 3 washes (15 min each) with saponin-based wash reagent. As negative control we used specimens not treated with EdU, but otherwise the specimens underwent all the procedures described above.

After visualization of the EdU labeling all the polyps and medusae were stained with the nuclei stain 4′, 6-diamidino-2-phenylindole (DAPI, 0.1 µg/ml, Life Technologies Europe BV, Nærum, Denmark) and mounted in Vectashield (Sigma-Aldrich, Brøndby, Denmark) on glass slides.

The specimens were scanned either on a laser-scanning confocal microscope (TCS SP2, Leica, Germany) or in a spinning-disc epifluorescent microscope (IX81, Olympus, Tokyo, Japan) and the final resolution of the images was set in Photoshop (CS 7.0.1., Adobe Systems).

### In vivo labeling of Tripedalia cystophora and Alatina moseri polyps and medusae

All specimens were starved for 24 to 36 h prior to EdU labeling.

Polyps: one hundred non-metamorphosing *T. cystophora* polyps were separated from the culture tank and placed in a Petri dish. Metamorphosis was induced by placing the dish in an incubation chamber and raising the temperature to 28°C. During the following two weeks the polyps in different stages of metamorphosis were collected for the labeling procedure. In the case of *A. moseri* the temperature was raised in the entire culture tank and polyps were collected for the labeling procedure directly from the tank. The metamorphosing and non-metamorphosing polyps of both species were incubated in 20 µM EdU in 500 µl sea water for 24 h at 28°C in 1.5 ml eppendorf tubes placed in the incubation chamber.

Juvenile medusae (1–3 days old): specimens were incubated in 20 µM EdU in 10 ml sea water for 8 h (*T. cystophora*) or 24 h (*A. moseri*) at 28°C in a Petri dish (θ = 6 cm) placed on the water surface in the culture tank.

Adult medusae: eight adult *T. cystophora* medusae were treated with 20 µM EdU in 80 ml sea water during daytime for 5 h at 28°C in a Petri dish (θ = 10 cm) placed on the water surface of the culture tank. Two of them were fixed right after EdU treatment (5 h). To trace the migration of marked S phase cells during one week we kept the other six medusae in a separate part of the culture tank. This separate chamber had a flow of water and medusae were fed daily. The size of the chamber allowed the treated medusae to swim and behave in their natural way. In pairs the medusae were fixed 24 h after EdU treatment (day 1), 72 h after treatment (day 3) and 168 h after treatment (day 7). Two rhopalia were removed from each of the eight jellyfish and sectioned on a Vibratome (VT1000s, Leica, Wetzlar, Germany) resulting in 50 µm sections which were then mounted in Elvanol on chromalun-coated glass slides.

Mid-sized medusae: four mid-sized (bell diameter  = 5–6 mm) medusae were treated with 20 µM EdU in 40 ml sea water for 5 h during the day (from 1100 hr till 1600 hr) at 28°C in a Petri dish (θ = 10 cm) placed on the water surface in the tank and four medusae were treated likewise at night (from 2200 hr till 0300 hr). Both groups had been fed in the morning the day before resulting in the medusae being starved for 24 or 36 h in daytime and nighttime experiments respectively. After the treatment the medusae underwent the EdU visualizing protocol. All the medusae were then dissected into quarters. Because of the tissue density in the rhopalium the scan could not reach the deeper cell layers and the jellyfish quarters, therefore, were then embedded in gelatin and sectioned on the Vibratome.

### Data analysis

The percentage of labeled S phase cells in the four main body parts of the juvenile medusae (the rhopalium, the pedalium, the manubrium including gastric filaments and the bell) was calculated from the ratio of EdU-stained cells to DAPI-stained cells. Cells were counted manually using Cell counter plugin of ImageJ software (ImageJ 1.46). To look for areas of higher proliferation rate, the data were tested in a one-way ANOVA followed by a Tukey-Kramer post hoc test. In the daytime vs. nighttime experiments the number of S phase cells in 200×200 µm area of the three predefined body parts (the bell area midways over the radial channel, a randomly chosen pedalium and a randomly chosen stalk base) and an entire rhopalium were counted and the two datasets from each body part were compared using unpaired, 2 sided student t-tests. In the chase experiments using fully grown rhopalia the number of S phase cells were counted from all sections of four whole rhopalia after 5 h, 1 day, 3 days, and 7 days. Results were compared using a one-way ANOVA followed by a Tukey-Kramer post hoc test. All statistical tests had a critical *P*-value of 0.05.

### TEM

Rhopalia dissected from the medusae by cutting the rhopalial stalk midways were fixed in 2.5% glutaraldehyde, 2.0% paraformaldehyde, and 3.0% sucrose in 0.15 M sodium cacodylate buffer. After 5–6 days in fixative they were washed twice in 0.15 M sodium cacodylate buffer and post fixed in 1% osmium tetroxide for 1 h at room temperature. They were then dehydrated in a series of ethanol, transferred to pure acetone, and embedded in Epon 812 resin. Sections of 50 nm thickness were made on an Ultracut microtome (UCT, Leica, Germany) and put on single slot grids. The sections were contrasted with 4% uranyl acetate for 20 min at room temperature and with 2% lead citrate for 5 min at 5°C.

## Results

### Proliferation zones changes during *Tripedalia cystophora* polyp metamorphosis

**Figure 2:**
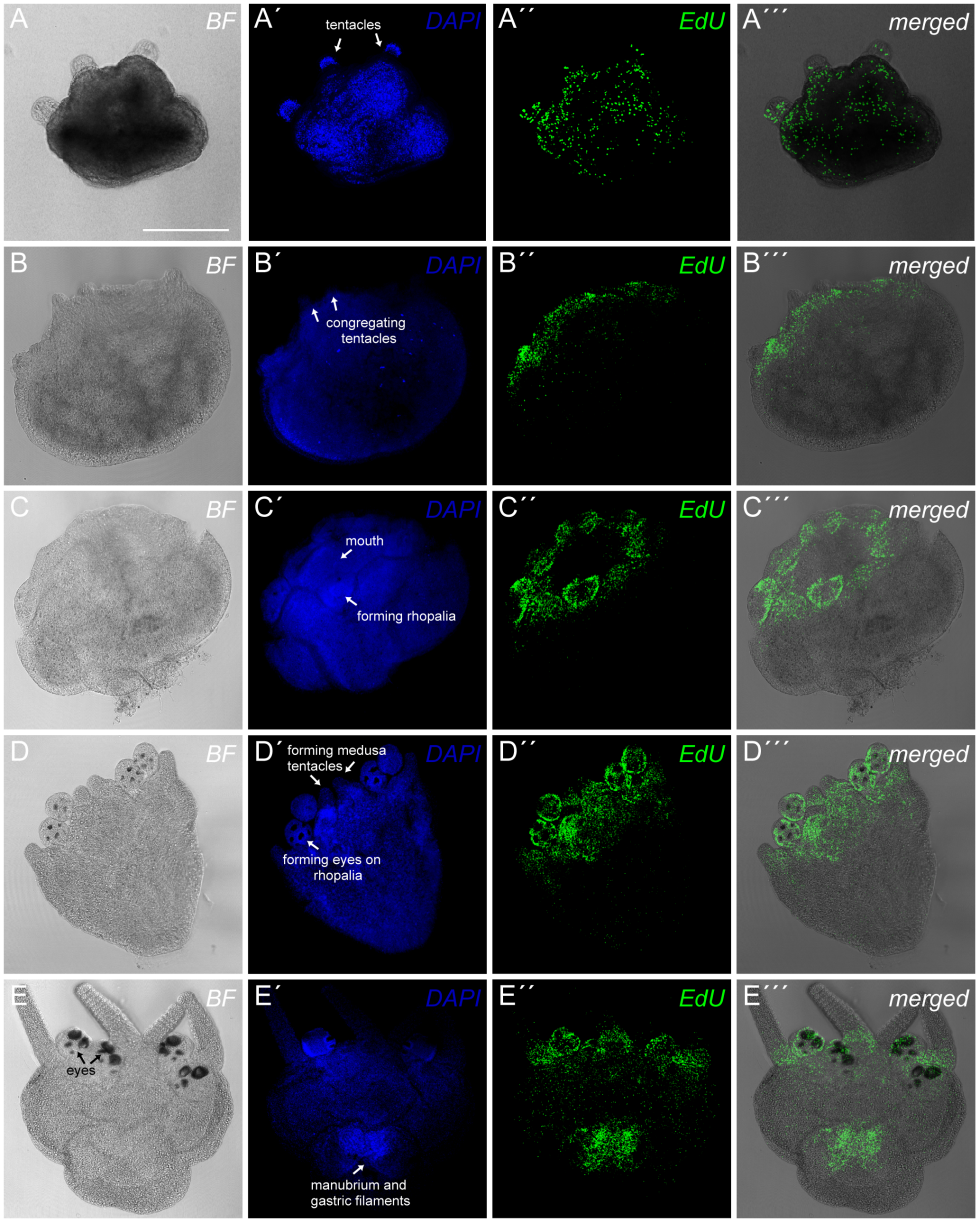
Proliferation zones during the metamorphosis of *Tripedalia cystophora* polyps. *T. cystophora* polyps during different stages of metamorphosis stained with DAPI (A′,B′, C′, D′, E′) and S phase cells visualized with EdU (A′′, B′′, C′′, D′′, E′′). (A-A′′′) Non-metamorphosing polyp. The S phase cells in non-metamorphosing polyps are dispersed throughout the entire body including the tentacles (A′′′). (B-B′′′) Polyp in the early stage of metamorphosis (stage of congregating tentacles). High density of S phase cells defining a proliferation zone is seen around the mouth and at the tentacle bases (B′′′). (C-C′′′) Metamorphosing polyp in the early stage of rhopalia formation. A proliferation zone is found in the oral end of the polyp and especially in the forming rhopalia (C′′′). (D-D′′′) Metamorphosing polyp in the late stage of rhopalia formation (forming eyes visible on the rhopalia). The highest number of S phase cells is again in the forming rhopalia but also in the growing medusa tentacles (D′′′). (E-E′′′) Polyp in the late stage of metamorphosis. An additional proliferation zone is observed in the forming manubrium and gastric filaments (E′′′). Scale bar, 300 µm (A) applies to all the pictures.

We found that in non-metamorphosing *T. cystophora* polyps the labeled S phase cells appear to be dispersed all over the body of the polyp including the tentacles (Figure 2A-A′′′). Immediately after metamorphosis starts, indicated by the polyp tentacles gathering in the four corners of the body (Figure 2B′), we observed clear proliferation zone in the area surrounding the mouth and in the proximal part of the tentacles (Figure 2B-B′′′). In the next step of the metamorphosis there were no longer labeled cells in the tentacles proper indicating that they have started to degenerate but their proximal part still constitutes a part of the proliferation zone marking the area where the rhopalia are being formed (Figure 2C-C′′′).

Later during metamorphosis when the eyes have become visible on the forming rhopalia (Figure 2D′) we still observed zones of high proliferation in the rhopalia and around the mouth where amongst other things the ring nerve is being reorganized [20] (Figure 2D-D′′′). In this stage the four primary tentacles of the future medusa also start to appear (Figure 2D′) indicated by clear proliferation zones between the rhopalia. In the last stage of the metamorphosis the polyp is in principle a medusa attached to the substrate by the apex of the bell (Figure 2E). At this stage the before mentioned proliferation zones prevailed, but additionally a high density of S phase cells was found in the gastrodermal part of the apical end of the animal (Figure 2E-E′′′). Here the manubrium, the mouth of the medusa, is being formed along with the gastric filaments. Further, at this stage, there was a seemingly uniform dispersal cell proliferation in the bell.

**Figure 3:**
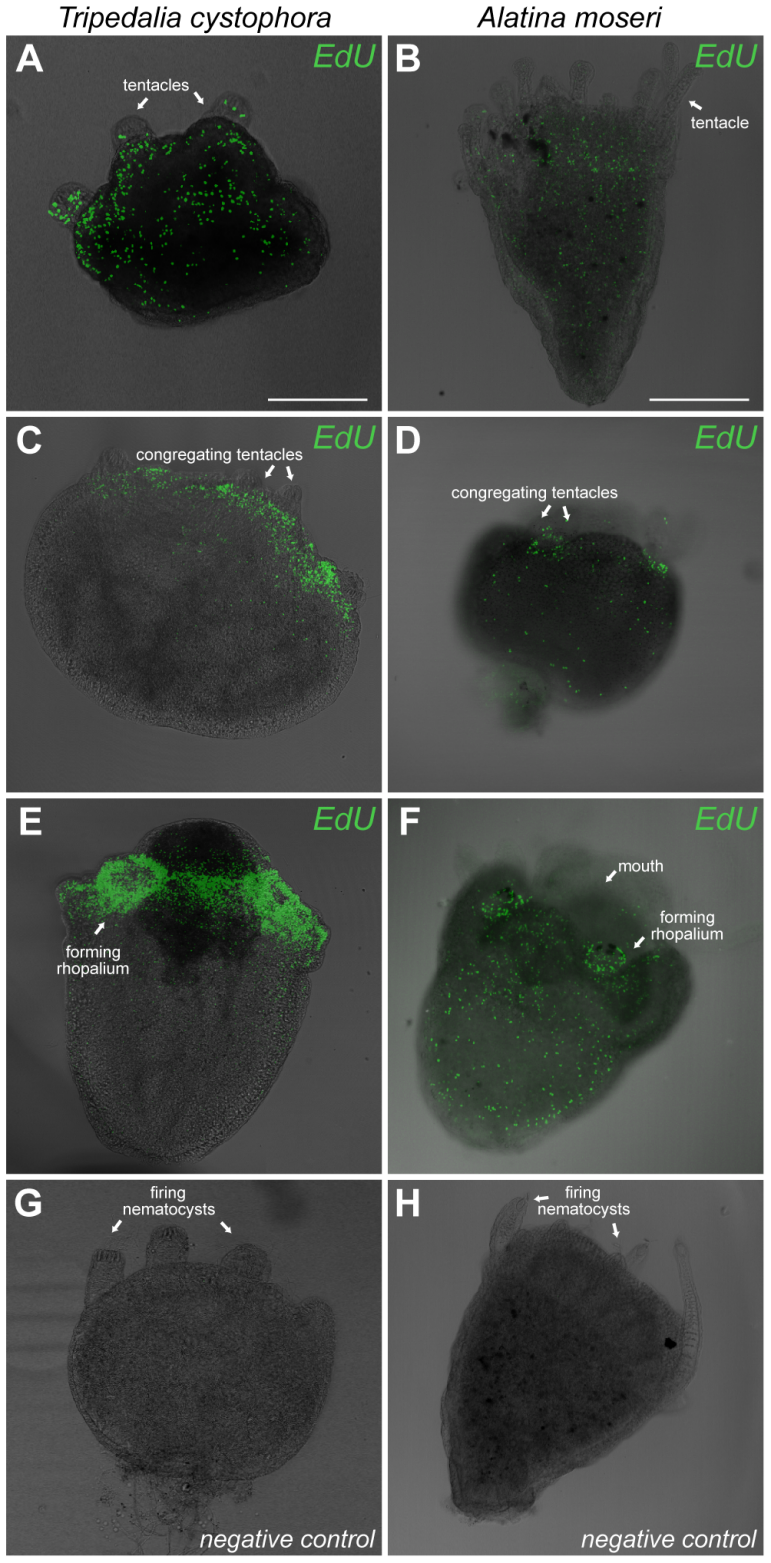
Comparison of proliferation zones during metamorphosis in *Tripedalia cystophora* and *Alatina moseri*. (A, B) Dispersed proliferation in the non-metamorphosing polyps. The overall pattern of proliferating cells is the same in two species with labeled cells seen throughout the body. (C, D) Both species show high S phase cells density in the early metamorphosis at the distal end of the body especially where the rhopalia are forming. Still, *T. cystophora* shows higher concentration of labeled cells (C). (E, F) The most apparent proliferation zone is found in the forming rhopalia and in the area surrounding the mouth where the ring nerve is reorganizing. At the end of the metamorphosis there are some differences between two species. Again *A. moseri* has fewer labeled S phase cells (F) than *T. cystophora*. It is also evident that the cells (at least the nuclei) are smaller in *T. cystophora* than in *A. moseri* (note the difference in scale bars). (G, H) Negative controls. Scale bars, 300 µm (A) applies to C, E, G; 600 µm (B) applies to D, F, H.

From gross morphological examinations there are indications that the metamorphosis is similar in all examined cubozoans [2], [3], [21]. Our data on proliferation patterns in *T. cystophora* and *A. moseri* confirms the similarity at least between these two species (Figure 3). We observed the same pattern in non-metamorphosing polyps with the S phase cells dispersed throughout the main body and tentacles (Figure 3A, B). When entering the metamorphosis, the proliferation zones from *T. cystophora* were also found in *A. moseri*: the area around the mouth, especially where the rhopalia and the new tentacles are formed (Figure 3C–F).

### Proliferation zones in juvenile cubozoan medusae

**Figure 4:**
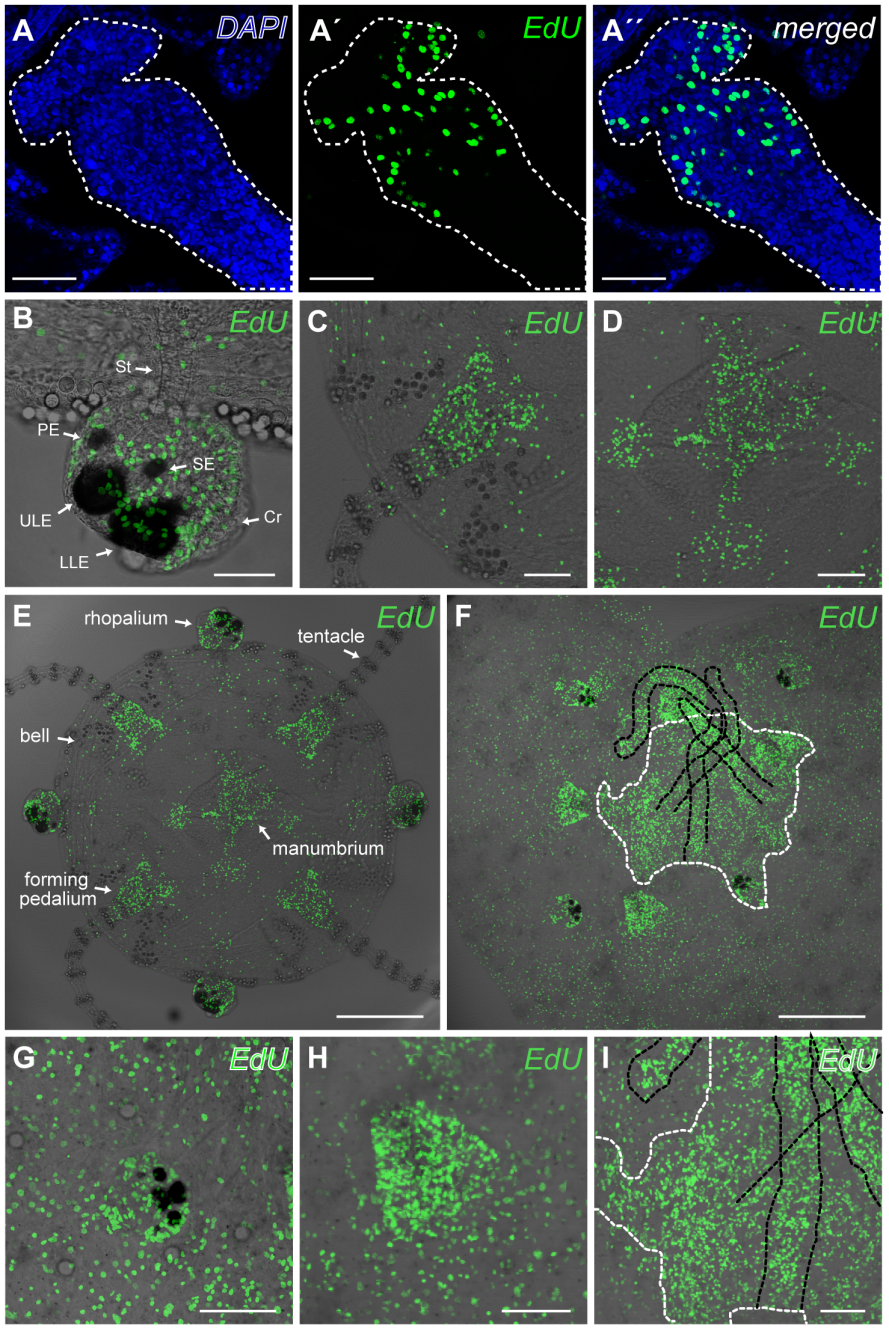
Proliferation zones in juvenile medusae of *Tripedalia cystophora* and *Alatina moseri*. (A-A′′) The marked area (white dashed line) indicates where DAPI-stained (A) and EdU-stained (A′) nuclei were counted in a pedalium of a juvenile medusa. (B–E) Proliferation zones in juvenile medusae of *T. cystophora*. The highest rate of proliferation is found in the rhopalia (B) and in the proximal part of the pedalia (before the first ring of nematocytes) (C). The rate of cell proliferation in the manubrium and gastric filaments (D) seems to be as high as in the rhopalia (B) and pedalia (C), but this is due to very high cell density in the manubrium. The proliferation rate is as low as in the bell (see also Table 1 and results section for details on statistics). (F–I) Proliferation zones in juvenile medusae of *A. moseri*. As seen for the polyps, the overall proliferation pattern is the same for two species, and proliferation zones are again found in the rhopalia (G) and pedalia (H). The manubrium (white dashed line) and gastric filaments (black dashed line) also display many S phase cells (I). PE represents pit eye; SE, slit eye; ULE, upper lens eye; LLE, lower lens eye; St, stalk; Cr, crystal. Scale bars, 50 µm (A, A′, A′′, B); 100 µm (C, D, G, H, I); 300 µm (E, F).

We followed the proliferation into the juvenile medusae (1–3 days old, bell diameter  = 1.0–1.2 mm, long axis of rhopalium  = 100–175 µm) of both species (Figure 4). The total number of the cells (Table S1) in the four areas of interest of *T. cystophora* was counted from the DAPI-stained cells and used to calculate the percentage of S phase cells (Table 1) (Figure 4A-A′′, Figure S2). The pedalium had the highest rate with 18.5% of the cells entering the S phase during the 8 h long incubation. The least were found in the bell were only about 1.9% of cells were labeled. The rhopalium and the pedalium (Figure 4B, C) both had significantly higher rates of proliferation than the manubrium and the bell (Figure 4D, E), (One-way ANOVA, F_(3,12)_ = 27.4, *P*<0.0001, followed by Tukey-Kramer post hoc Test, 0.0001<*P*<0.0058). There was no difference between the rhopalium and pedalium or between the manubrium and bell (One-way ANOVA, F_(3,12)_ = 27.4, *P*<0.0001, followed by Tukey-Kramer post hoc test, *P* = 0.062 and 0.83 respectively). The micrographs indicate that the manubrium (Figure 4D) also constitutes a zone with enhanced proliferation rate, but the results showed that this is due to the fact that the cells here are in general small and the cell density is therefore higher when compared to the bell (8.4 cells/100 µm^2^ in the manubrium vs. 0.6 cell/100 µm^2^ in the bell). In order to check if the bell is growing by cell enlargement instead we compared the cell density in the bell of juvenile, sub-adult and adult medusa (Figure S3). We found that the cell density in the bell increased with age: 0.62 cell/100 µm^2^ in juveniles, 0.85 cell/100 µm^2^ in sub-adult medusa and 1.76 cell/100 µm^2^ in adult medusa.

**Table 1:** Percentage of S phase cells in predefined areas of juvenile *Tripedalia cystophora* (see also Figure 4, Figure S2 and Table S1).

predefined area	% S phase cells *
rhopalium	13.0±0.3
pedalium	18.5±3.0
manubrium	3.6±0.5
bell	1.9±0.4

* values are means ± S.E.M, *n* = 4.

### Proliferation in adult *Tripedalia cystophora* rhopalia

**Figure 5:**
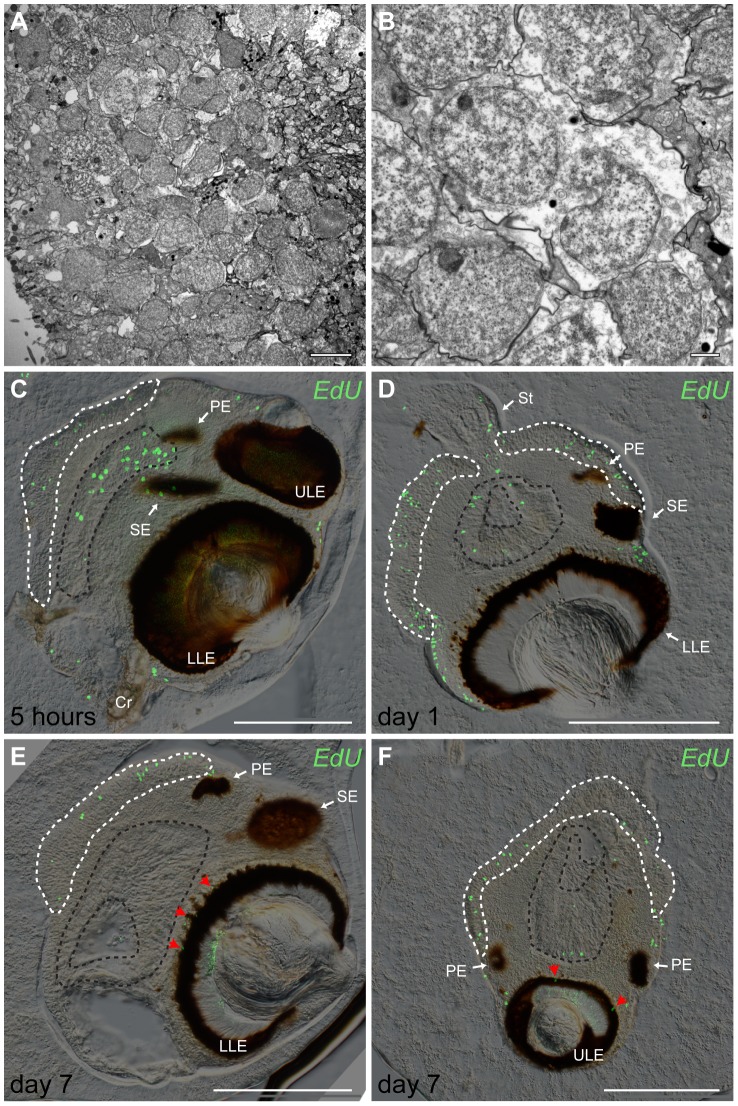
Cell turnover in adult *Tripedalia cystophora* rhopalia. (A, B) TEM pictures of the cells in posterior cell sheet. These cells have typical morphology of undifferentiated cells (small nuclei, little cytoplasm) (A). Dividing cells in the posterior cell sheet (B). (C) Vertical peripheral section of adult rhopalium. Labeled cells are after 5 h localized in the posterior cell sheet (white dashed area), gastrodermis (black dashed area) and epidermis. (D) Horizontal central section through LLE of rhopalium. Labeled cells, 24 h (day 1) after treatment, do not change localization and are still observed in the posterior cell sheet (white dashed line), gastrodermis (black dashed line) and epidermis. (E, F) Within one week labeled S phase cells migrated into the retinas of ULE (E, red arrowheads) and LLE (F, red arrowheads). (E) Vertical central section of an adult rhopalium. (F) Horizontal peripheral section through ULE of an adult rhopalium. PE represents pit eye; SE, slit eye; ULE, upper lens eye; LLE, lower lens eye; St, stalk; Cr, crystal. Scale bars, 5 µm (A); 1 µm (B); 200 µm (C–F).

We also examined the proliferation in fully grown rhopalia (medusa diameter  = 9–9.5 mm, long axis of rhopalium  = 500–600 µm) of *T. cystophora* (Figure 5). We mapped the location of S phase cells and counted their numbers in the entire rhopalium just after incubation with EdU (5 h) (Figure 5C) and again after 24 h (day 1), 72 h (day 3) and 168 h (day 7) (Figure 5D–F). After 5 h all the marked cells were located either in the posterior cell sheet (Figure 5C, white dashed area), in the gastrodermis (Figure 5C, black dashed area) or in the epithelium of the rhopalium. After both 72 and 168 h there were still labeled cells in the original positions but additional labeled cells were found in association with the retinas of the lens eyes (Figure 5E, F, red arrowheads). Surprisingly, there was a tendency of a decline in the number of marked cells over time (Table 2). The differences were not significant though (One-way ANOVA, F_(3,12)_ = 3.3, *P* = 0.06, followed by Tukey-Kramer post hoc Test, 0.14<*P*<0.99).

**Table 2:** Counts of S phase cells in fully grown rhopalia of *Tripedalia cystophora* after 5, 24, 72 and 168 h (see also Figure 5).

timescale	count of S phase cells *
5 hours	382±141
24 hours	374±130
72 hours	129±29
168 hours	96±31

* values are means ± S.E.M, *n* = 4.

### Diurnal pattern in the proliferation in *Tripedalia cystophora*

**Figure 6:**
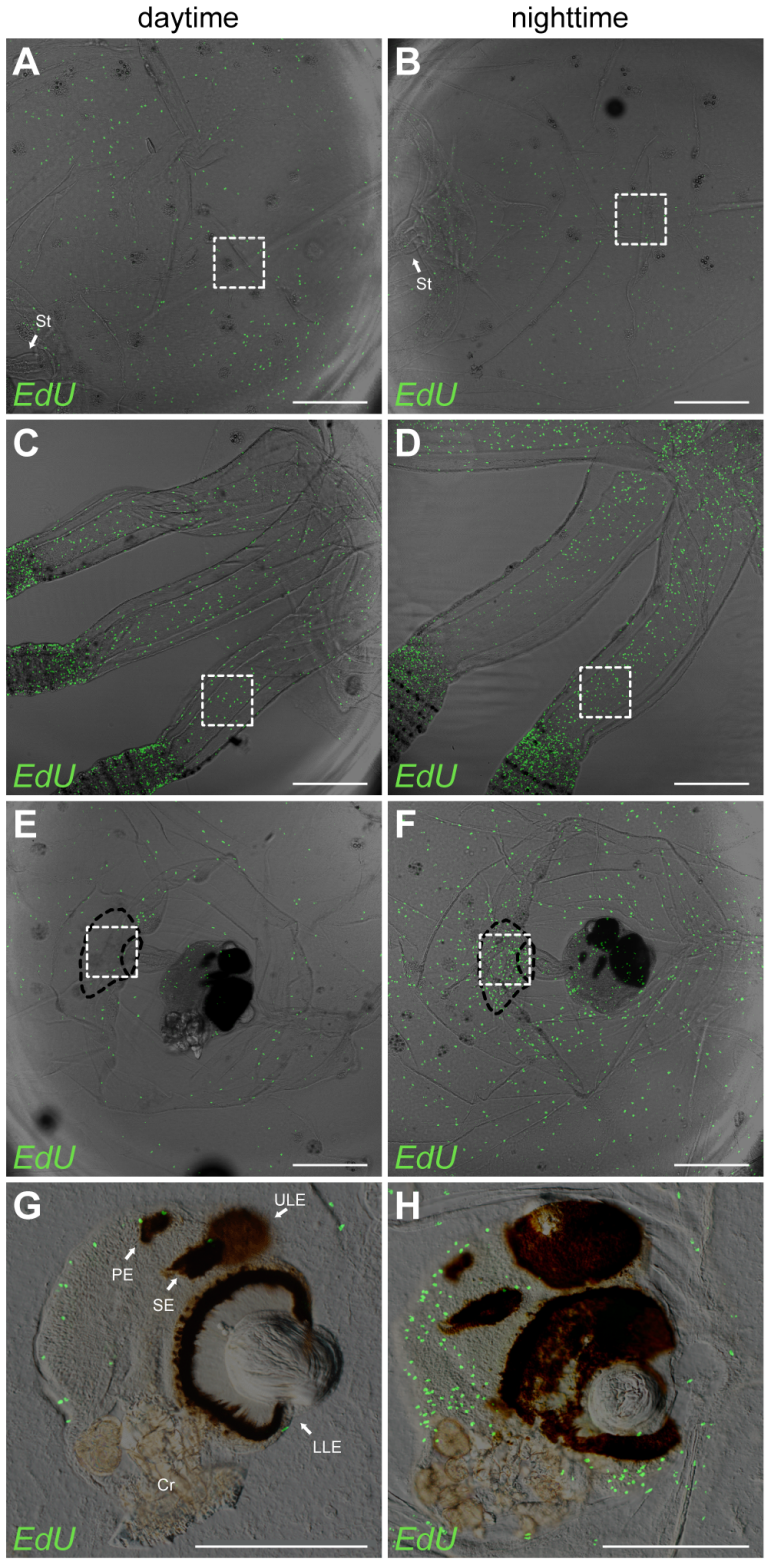
Diurnal change in the rate of proliferation in *Tripedalia cystophora*. (A, C, E, G) Proliferation during daytime in the bell area midways over the radial channel (A), in the pedalium (C), at the stalk base (E) and in the rhopalium (G). The same areas were likewise labeled during nighttime (B, D, F, H). White dashed squares indicate areas of cell counting (200×200 µm). Black dashed area indicates where the ring nerve enters rhopalial stalk (stalk base). The proliferation in the pedalium (D), stalk base (F) and rhopalium (H) is significantly higher at night than during the day (see result section for details on statistics). PE represents pit eye; SE, slit eye; ULE, upper lens eye; LLE, lower lens eye; Cr, crystal. Scale bars, 300 µm (A–F); 200 µm (G, H).

The number of cells entering the S phase at night and during the day was compared in four predefined areas of the medusa. The four areas were 1) 200×200 µm of the bell area midways on the top of the radial channel (Figure 6A, B, white dashed square), 2) 200×200 µm midways on the pedalium (Figure 6C, D, white dashed square), 3) 200×200 µm at the stalk base (Figure 6E, F, white dashed square) where the ring nerve enters the stalk (Figure 6E, F, black dashed area), and 4) the entire rhopalium (long axis of rhopalium  = 350–450 µm) (Figure 6G, H). We found that the number of S phase cells in the pedalium, the stalk base, and the rhopalia was higher during nighttime for each of these areas in all the samples (Table S2). The averages of these cell counts were significantly higher during nighttime than during daytime (Table 3) (student t-test, 2 sided, non-paired, *P* = 0,006, *P* = 0,006 and *P* = 0,008, respectively, *n* = 4). In the case of the stalk base we found five times more cells in the S phase at night. In the bell half the samples had the most labelled cells during the day and half during the nighttime (Table S2) and the difference between the averages was not statistically significant (student t-test, 2 sided, non-paired, *P* = 0.23, *n* = 4) (Table 3).

**Table 3:** Counts of S phase cells at daytime and nighttime in predefined area of different body parts of *Tripedalia cystophora* (see also Figure 6 and Table S2).

body part	count of S phase cells *		student t-test
	daytime	nighttime	*P* - value
bell	11±1	23±10	0.2
pedalium	13±5	45±8	0.006
stalk base	14±6	75±16	0.006
rhopalium	94±10	253±46	0.008

* values are means ± S.E.M, *n* = 4.

## Discussion

Using *in vivo* labeling we have marked the S phase cells and described proliferation patterns in polyps and medusae of the cubozoans *T. cystophora* and *A. moseri*. We have focused on the reorganization, formation and growth of especially the CNS. We show that when the polyp metamorphoses into the juvenile medusa, the new forming structures are associated with zones of enhanced proliferation, indicating that they are formed *de novo*; still we cannot completely rule out a possible role of reorganization and/or redifferentiation of already existing cells. A high rate of proliferation is found in some body parts (pedalia and rhopalia) in all the examined life stages including adult medusae where growth has arrested. This suggests that these areas which include a large part of the CNS have a high rate of cell turnover.

### Proliferation zones in cubozoan polyps

During their lifecycle cubozoans undergo two metamorphoses: first when they metamorphose from larva into primary polyp and second when the polyp becomes a free swimming medusa (Figure 1). Here we have examined the latter. The previous knowledge on metamorphosing polyps in cubozoans stems entirely from descriptions on the gross morphological level of the process [2], [3], [8], [20]–[22]. We went further and looked at some of the details using *in vivo* labeling of S phase cells [18] and described proliferation zones in metamorphosing polyps of *T. cystophora* and *A. moseri*. Cell proliferation appears to be distributed throughout the body including the tentacles in non-metamorphosing polyps (Figure 2A′′′). This is comparable with the results from the other cnidarian polyps: *Aiptasia diaphana*, *Nematostella vectensis* as well as in *Aurelia aurita*, where dividing cells were also found in the entire polyp including the tentacles [23]–[25].

Profound changes of proliferation in *T. cystophora* polyps were observed at the onset of the metamorphosis when complex structures like rhopalia, tentacles, manubrium, ring nerve and gastric filaments develop. The identification of proliferation zones in many of the above mentioned structures was not unexpected, since these body parts need to be formed *de novo* to form the new medusa. The ring nerve constitutes the largest part of the CNS in cubozoans, and curiously when becoming a medusa it loses its gastrodermal part and greatly expands the epidermal part [26], [27].

In the polyps of *A. moseri* we observed a slightly different pattern of the proliferation. The overall proliferation zones were the same but we still found proliferating cells in the tentacles of *A. moseri* polyps at the stage when rhopalia are forming (Figure 3D). This suggests that even though the tentacles disappear not all their cells degenerate in this species, but some of them are probably being reprogrammed and obtain new functions in the forming medusa. The importance of this probable reuse needs future experiments with dedifferentiating and apoptosis markers to be evaluated.

It should be noted, that the species examined to date belong to the same clade (Carybdids) within Cubozoa and data from the other clade (Chirodropids) like *Chironex fleckeri* is called for in order to better embrace the diversity of the metamorphosis in cubopolyps.

### Proliferation zones in juveniles

We quantified the proliferation rate in the rhopalia, pedalia, the manubrium including the gastric filaments and in the bell of juvenile medusae of *T. cystophora*. We found the highest proliferation rate in the pedalium (Figure 4C) where 18.5% of the cells entered the S phase during the 8 h incubation. This fast growth matches the observation that in juvenile *T. cystophora* the tentacles grow rapidly including the addition of a new tentacle on each side of the primary ones. A high proliferation rate in the tentacle bulbs has also been found in another cnidarian *Podocoryne carnea* (Hydrozoa) [28]. Interestingly, in *T. cystophora* this zone of enhanced proliferation stops at the first nematocyte battery with only few labeled cells seen between the first and the second ring of nematocytes, and almost no S phase cells in the rest of the tentacle. There are two possible explanations for this pattern of forming new cells: either the complete nematocytes batteries are being formed in the pedalium including nematocytes progenitor cells and the tentacle is growing solely by adding these rings at the proximal part of tentacle, or the nematocyte precursors are formed in the pedalium and then migrate out through the tentacle to the more distal nematocyte batteries where they differentiate. The latter explanation seems more likely since the place of nematogenesis in the hydrozoan medusa *Clytia hemisphaerica* was reported to be in the tentacle bulb. A flow of the mitotic cells from the tentacle bulb then disperses them throughout the whole tentacle [29].

We found another proliferation zone in the rhopalia (Figure 4B) where 13.0% of the rhopalial cells entered the S phase within 8 h. This proliferation rate indicates rapid cell turnover in the rhopalium, since the relative growth of the rhopalia follows that of the bell where only about 1.9% of the cells entered S phase, which also have to compensate for the shown increase in cell density (Figure S3). This suggested high cell turnover is interesting, not least as the rhopalia holds a major part of the CNS. Thus, it becomes important to know the future of the new cells, e.g. whether they differentiate into nerve cells constantly replacing parts of the CNS. Such a system is indicated in the CNS of another cnidarian, *Hydra oligans* [16].

### Cell proliferation in fully grown rhopalia

Tissue homeostasis is an important physiological phenomenon that ensures a dynamic balance between cell proliferation and cell death during maintenance in all multicellular organisms. Progenitor cells are continuously recruited to differentiate into wanted specific cell types while damaged or unwanted cells are eliminated mostly through programmed cell death (apoptosis) [30].

Once the medusae of *T. cystophora* become sexually mature they stop feeding and arrest their growth (personal observations). However, at this stage we still observed a high rate of proliferation in the rhopalia supporting the above suggestion that if the cells complete the initiated cell cycle resulting in division they are mainly supporting cell turnover. Under the assumption that the same tissue of same sized animals have similar cell densities we have used literature data to estimate that the total number of cells in an adult rhopalium excluding the gastrodermis is approximately 13500 (1000 nerve cells, 2000 cells of posterior cell sheet [17], 2500 photoreceptors [31], [32] and considering the average size of an adult rhopalium being 400×600 µm with an average diameter of the epithelial cells of 8 µm, the estimate of the number of epithelial cells is around 8000). We found an average of 520 labeled cells after the 5 h of incubation in the adult rhopalium (excluding the gastrodermis) which is about 4% of all the cells. This crude estimate provides a preliminary insight into the rhopalial cell turnover which has to be further investigated using apoptosis markers.

Our findings support the hypothesis, that the posterior cell sheet is an area of cell division. In the rhopalia of adult *T. cystophora* medusae marked cells occurred after initial labeling in the posterior cell sheet, gastrodermis and epithelium only. To test if some of these cells are incorporated into the RNS or the eyes, we followed them for one week post-incubation. 24 h post-incubation the location of the labeled S phase cells had not changed (Figure 5D), but after both 72 and 168 h we found labeled cells further inside the rhopalium in association with the retinas of the lens eyes (Figure 5E, F, red arrowheads). This proves that at least some of the new cells differentiate into nerve cells, either photoreceptors or retina associated neurons, which are the only cells found in the retina [17]. Whether they originate from the posterior cell sheet is not known, though.

Interestingly, there is an indication of a decline in the number of labeled cells over time. After 168 h about one third of the originally labeled cells were still found in the posterior cell sheet. We hypothesize that after cell division one of the daughter cells stays behind in the posterior cell sheet and the other differentiates and migrates into the retina or other parts of rhopalium. The fact that we only observed a small number of labeled S phase cells in the retina again suggests a high cell turnover in rhopalium, but as mentioned before, this has to be further investigated using apoptosis and/or cell death markers to draw any final conclusions.

### Difference in day and nighttime proliferation in *Tripedalia cystophora*

Our results strongly suggest that in *T. cystophora* there is a diurnal pattern of proliferation in the pedalium, stalk base and rhopalium. In the bell, the observed difference between day and night proliferation patterns was not statistically significant. For practical reasons (the medusae only feed in light) we could not starve the medusae for the exact same period of time (24 vs 36 h) and it is not possible, therefore, to completely exclude that the diurnal pattern is caused by this difference. Diurnal rhythms in cell division has previously been demonstrated to occur from unicellular organisms to humans [33]. Further, in mammals the diurnal rhythm in proliferation can also be confined to some tissue specific cells [34].

Two of the areas of enhanced nocturnal proliferation, the rhopalia and stalk base, are in close connection with parts of the CNS. We speculate that this is due to the suggested cell turnover in the CNS mostly being initiated at night where little sensory input needs processing. Still, we do not know whether the labeled S phase cells will successfully finish the cell cycle resulting in their division and subsequent differentiation or whether they stop at the S/G2 phase cell cycle check point for a longer period of time. Even if they continue the cell cycle and differentiate into new nerve cells this process will probably take a few days [35], [36], which means that the coupling we see between proliferation and resting does not necessarily mean that possible change/renewal of the CNS happens at night.

## Supporting Information

Figure S1 **Top view of proliferation zones during metamorphosis of** ***Tripedalia cystophora*** **polyp.** *T. cystophora* polyp during different stages of metamorphosis stained with DAPI (A′, B′, C′, D′, E′) and S phase cells visualized with EdU (A′′, B′′, C′′, D′′, E′′). (A-A′′′) Non-metamorphosing polyp showing dispersed S phase cells in oral pole, tentacles and body. (B-B′′′) At the beginning of metamorphosis, in the stage of congregating tentacles, the shape of the oral pole is clearly circular (B′). Four proliferation zones can be observed at the bases of the tentacles marking the areas of the forming rhopalia (B′′′). (C-C′′′) The shape of the oral pole has changed into quadrangular (C′) and the proliferation zone is expanding in the developing rhopalia and the area surrounding the mouth (C′′′). (D-D′′′) Proliferation zones still prevail in the rhopalia, which now have developing eyes, and in the area surrounding the mouth (D′′′). (E-E′′′) In the last stage of metamorphosis an additional proliferation zone is observed in the forming manubrium including the gastric filaments. S phase cells are distributed in the bell of the future medusa (E′′′). Scale bar, 300 µm (A) applies to all the pictures.
(TIF)

Figure S2 **Micrographs used to calculate the percentage of S phase labeled cells.** Four different body parts of juvenile medusae stained with DAPI (A–D) and EdU (A′-D′) in order to calculate the proliferation rates. 10 µm thick confocal stacks used for cell counts of DAPI- and EdU-stained cells in bell (A-A′′), manubrium (B-B′′), pedalium (C-C′′) and rhopalium (D-D′′). White dashed line indicates the area of cell counts. Scale bar, 50 µm (A) applies to all the pictures.
(TIF)

Figure S3 **Cell density in the bell of** ***Tripedalia cystophora*** **changes with the age of the medusa.** Bell of a juvenile, sub-adult and adult *T. cystophora* medusa stained with DAPI. The area of 100×100 µm used for nuclei counts in the bell of juvenile (A), sub-adult (B) and adult medusae (C). Scale bar, 20 µm (A) applies to all the pictures.
(TIF)

Table S1 **Counts of DAPI and EdU labeled cells in predefined areas of four different body parts of juvenile medusa of** ***Tripedalia cystophora*** **.** Ratio of numbers of EdU to DAPI labeled cells results in the percentage of the S phase cells in the given body part (see also Figure S2).
(DOCX)

Table S2 **Counts of EdU labeled cells in predefined areas of 200×200 µm of four different body parts of mid-sized medusa** ***Tripedalia cystophora*** **during daytime and during nighttime (see also** **Figure 6** **).**
(DOCX)

## References

[1] Collins AG (2002) Phylogeny of Medusozoa and the evolution of cnidarian life cycles. J Evol Biol 15: 418–432.

[2] Straehler-Pohl I, Jarms G (2005) Life cycle of Carybdea marsupialis Linnaeus, 1758 (Cubozoa, Carybdeidae) reveals metamorphosis to be a modified strobilation. Mar Biol 147: 1271–1277.

[3] Werner B, Cutress CE, Studebaker JP (1971) Life cycle of Tripedalia cystophora Conant (Cubomedusae). Nature 232: 582–583. 16063105 10.1038/232582a0

[4] Claus C (1878) Ueber Charybdea marsupialis. Arb Zool Inst Universität Wien 1: 1–56.

[5] Conant FS (1898) The Cubomedusae. Mem Biol Lab Johns Hopkins Univ 4: 1–61.

[6] Berger EW (1900) Physiology and histology of Cubomedusae, including Dr. F.S. Conant′s notes on the physiology. Mem Biol Lab Johns Hopkins Univ 4: 1–84.

[7] Yamasu T, Yoshida M (1976) Fine structure of complex ocelli of a cubomedusan, Tamoya bursaria Haeckel. Cell Tissue Res 170: 325–339. 8210 10.1007/BF00219415

[8] Laska G, Hündgen M (1982) Morphologie und Ultrastruktur der Lichtsinnesorgane von Tripedalia cystophora Conant (Cnidaria, Cubozoa). Zool Jb Anat 108: 107–123.

[9] Garm A, Oskarsson M, Nilsson DE (2011) Box jellyfish use terrestrial visual cues for navigation. Curr Biol 21: 798–803. 21530262 10.1016/j.cub.2011.03.054

[10] Buskey E (2003) Behavioral adaptations of the cubozoan medusa Tripedalia cystophora for feeding on copepod (Dioithona oculata) swarms. Mar Biol 142: 225–232.

[11] Garm A, O'Connor M, Parkefelt L, Nilsson DE (2007) Visually guided obstacle avoidance in the box jellyfish Tripedalia cystophora and Chiropsella bronzie. J Exp Biol 210: 3616–3623. 17921163 10.1242/jeb.004044

[12] Garm A, Ekstrom P, Boudes M, Nilsson DE (2006) Rhopalia are integrated parts of the central nervous system in box jellyfish. Cell Tissue Res 325: 333–343. 16557386 10.1007/s00441-005-0134-8

[13] Parkefelt L, Skogh C, Nilsson DE, Ekstrom P (2005) Bilateral symmetric organization of neural elements in the visual system of a coelenterate, Tripedalia cystophora (Cubozoa). The Journal of comparative neurology 492: 251–262. 16217792 10.1002/cne.20658

[14] Mackie GO (2004) Central neural circuitry in the jellyfish Aglantha: a model 'simple nervous system'. Neuro-Signals 13: 5–19. 15004422 10.1159/000076155

[15] Mackie GO (2008) Immunostaining of peripheral nerves and other tissues in whole mount preparations from hatchling cephalopods. Tissue & cell 40: 21–29. 17953977 10.1016/j.tice.2007.08.005

[16] Koizumi O, Itazawa M, Mizumoto H, Minobe S, Javois LC, et al (1992) Nerve ring of the hypostome in hydra. I. Its structure, development, and maintenance. J Comp Neurol 326: 7–21. 1479070 10.1002/cne.903260103

[17] Skogh C, Garm A, Nilsson DE, Ekstrom P (2006) Bilaterally symmetrical rhopalial nervous system of the box jellyfish Tripedalia cystophora. Journal of morphology 267: 1391–1405. 16874799 10.1002/jmor.10472

[18] Salic A, Mitchison TJ (2008) A chemical method for fast and sensitive detection of DNA synthesis in vivo. Proc Natl Acad Sci U S A 105: 2415–2420. 18272492 10.1073/pnas.0712168105 PMC2268151

[19] Garm A, Bielecki J, Petie R, Nilsson DE (2012) Opposite patterns of diurnal activity in the box jellyfish Tripedalia cystophora and Copula sivickisi. Biol Bull 222: 35–45. 22426630 10.1086/BBLv222n1p35

[20] Laska-Mehnert G (1985) Cytologishe veränderungen während der metamorphose des cubopolypen Tripedalia cystophora (Cubozoa, Carybdeidae) in die medusae. Helgoländer wiss Meeresunters 39: 129–164.

[21] Arneson AC, Cutress CE (1976) Life history of *Carybdea alata* Reynaud, 1831 (Cubomedusae). In: Coelenterate Ecology and Behavior (G. O Mackie, ed.). Plenum Press, New York: 227–236.

[22] Laska G, Hündgen M (1984) Die ultrastruktur des neuromuskulären systems der medusen von Tripedalia cystophora und Carybdea marsupialis (Coelenterata, Cubozoa). Zoomorphology 104: 163–170.

[23] Singer II (1971) Tentacular and oral-disc regeneration in the sea anemone, Aiptasia diaphana. 3. Autoradiographic analysis of patterns of tritiated thymidine uptake. Journal of embryology and experimental morphology 26: 253–270. 4400497

[24] Passamaneck YJ, Martindale MQ (2012) Cell proliferation is necessary for the regeneration of oral structures in the anthozoan cnidarian Nematostella vectensis. BMC developmental biology 12: 34. 23206430 10.1186/1471-213X-12-34 PMC3553063

[25] Gold DA, Jacobs DK (2013) Stem cell dynamics in Cnidaria: are there unifying principles? Development genes and evolution 223: 53–66. 23179637 10.1007/s00427-012-0429-1 PMC7211294

[26] Satterlie RA (2002) Neuronal control of swimming in jellyfish: a comparative story. Can J Zool 80: 1654–1669.

[27] Chapman DM (1978) Microanatomy of the cubopolyp, Tripedalia cystophora (Class Cubozoa). Helgoländer wiss Meeresunters 31: 128–168.

[28] Spring J, Yanze N, Middel AM, Stierwald M, Groger H, et al (2000) The mesoderm specification factor twist in the life cycle of jellyfish. Developmental biology 228: 363–375. 11112336 10.1006/dbio.2000.9956

[29] Denker E, Manuel M, Leclere L, Le Guyader H, Rabet N (2008) Ordered progression of nematogenesis from stem cells through differentiation stages in the tentacle bulb of Clytia hemisphaerica (Hydrozoa, Cnidaria). Developmental biology 315: 99–113. 18234172 10.1016/j.ydbio.2007.12.023

[30] Green DR (2011) Means to an end: Apoptosis and other cell death mechanisms. Cold Spring Harbor Laboratory Press.

[31] Nilsson DE (2005) Photoreceptor evolution: ancient siblings serve different tasks. Current biology: CB 15: R94–96. 15694299 10.1016/j.cub.2005.01.027

[32] Ekstrom P, Garm A, Palsson J, Vihtelic TS, Nilsson DE (2008) Immunohistochemical evidence for multiple photosystems in box jellyfish. Cell and tissue research 333: 115–124. 18504619 10.1007/s00441-008-0614-8

[33] Johnson CH (2010) Circadian clocks and cell division: what's the pacemaker? Cell cycle 9: 3864–3873. 20890114 10.4161/cc.9.19.13205 PMC3047750

[34] Biederbick A, Elsasser H (1998) Diurnal pattern of rat pancreatic acinar cell replication. Cell and tissue research 291: 277–283. 9426314 10.1007/s004410050997

[35] Campbell RD, David CN (1974) Cell cycle kinetics and development of Hydra attenuata. II. Interstitial cells. Journal of cell science 16: 349–358. 4448825 10.1242/jcs.16.2.349

[36] David CN, Campbell RD (1972) Cell cycle kinetics and development of Hydra attenuata. I. Epithelial cells. Journal of cell science 11: 557–568. 5076361 10.1242/jcs.11.2.557

