# Supplementary figures and images for: Cell Proliferation in Cubozoan Jellyfish Tripedalia cystophora and Alatina moseri
Source: PLoS One. 2014 Jul 21;9(7):e102628. doi: 10.1371/journal.pone.0102628 (PMC4105575; doi:10.1371/journal.pone.0102628)

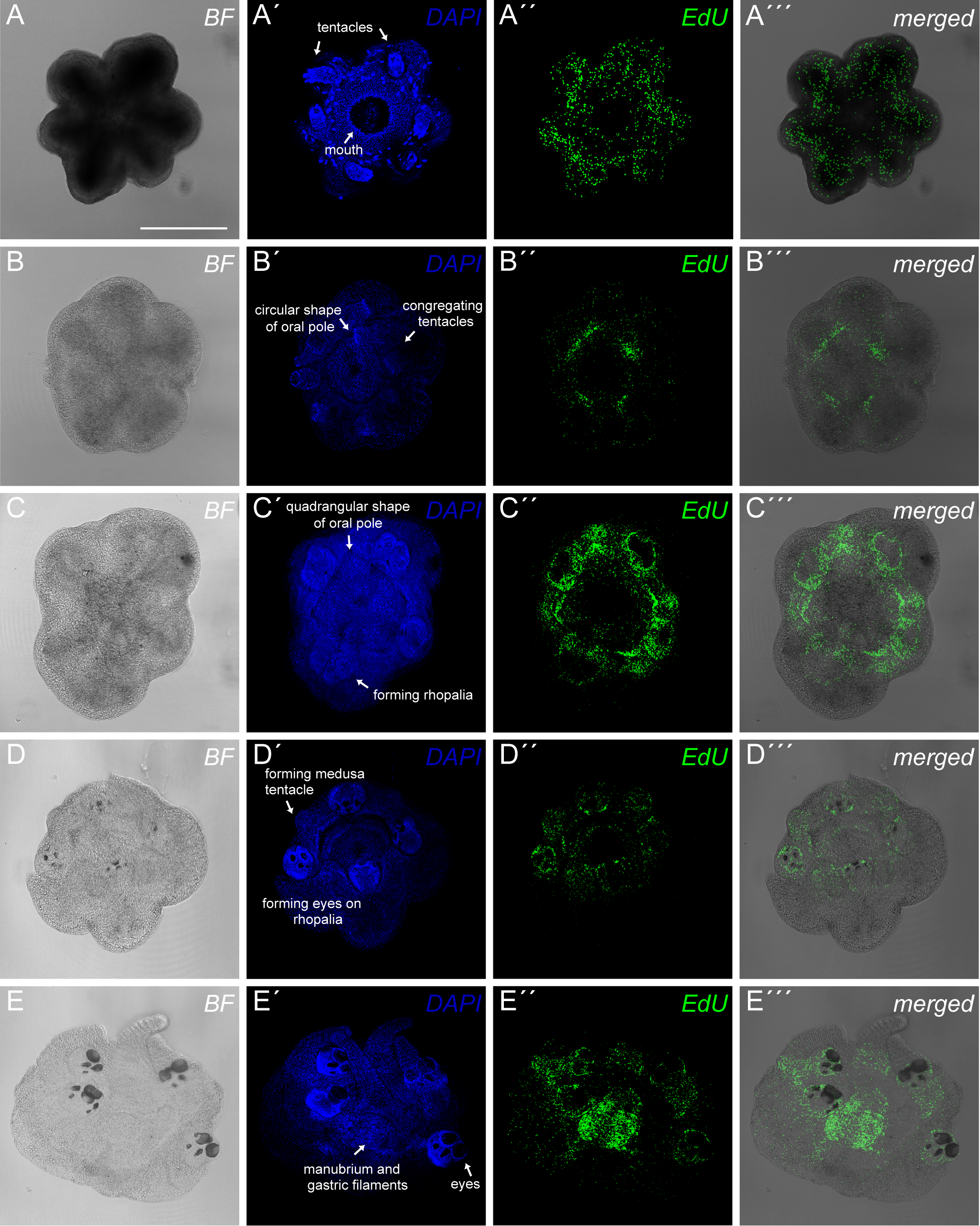

Supplement: Figure S1 — Top view of proliferation zones during metamorphosis of Tripedalia cystophora polyp. T. cystophora polyp during different stages of metamorphosis stained with DAPI (A′, B′, C′, D′, E′) and S phase cells visualized with EdU (A′′, B′′, C′′, D′′, E′′). (A-A′′′) Non-metamorphosing polyp showing dispersed S phase cells in oral pole, tentacles and body. (B-B′′′) At the beginning of metamorphosis, in the stage of congregating tentacles, the shape of the oral pole is clearly circular (B′). Four proliferation zones can be observed at the bases of the tentacles marking the areas of the forming rhopalia (B′′′). (C-C′′′) The shape of the oral pole has changed into quadrangular (C′) and the proliferation zone is expanding in the developing rhopalia and the area surrounding the mouth (C′′′). (D-D′′′) Proliferation zones still prevail in the rhopalia, which now have developing eyes, and in the area surrounding the mouth (D′′′). (E-E′′′) In the last stage of metamorphosis an additional proliferation zone is observed in the forming manubrium including the gastric filaments. S phase cells are distributed in the bell of the future medusa (E′′′). Scale bar, 300 µm (A) applies to all the pictures. (TIF) [file pone.0102628.s001.tif]

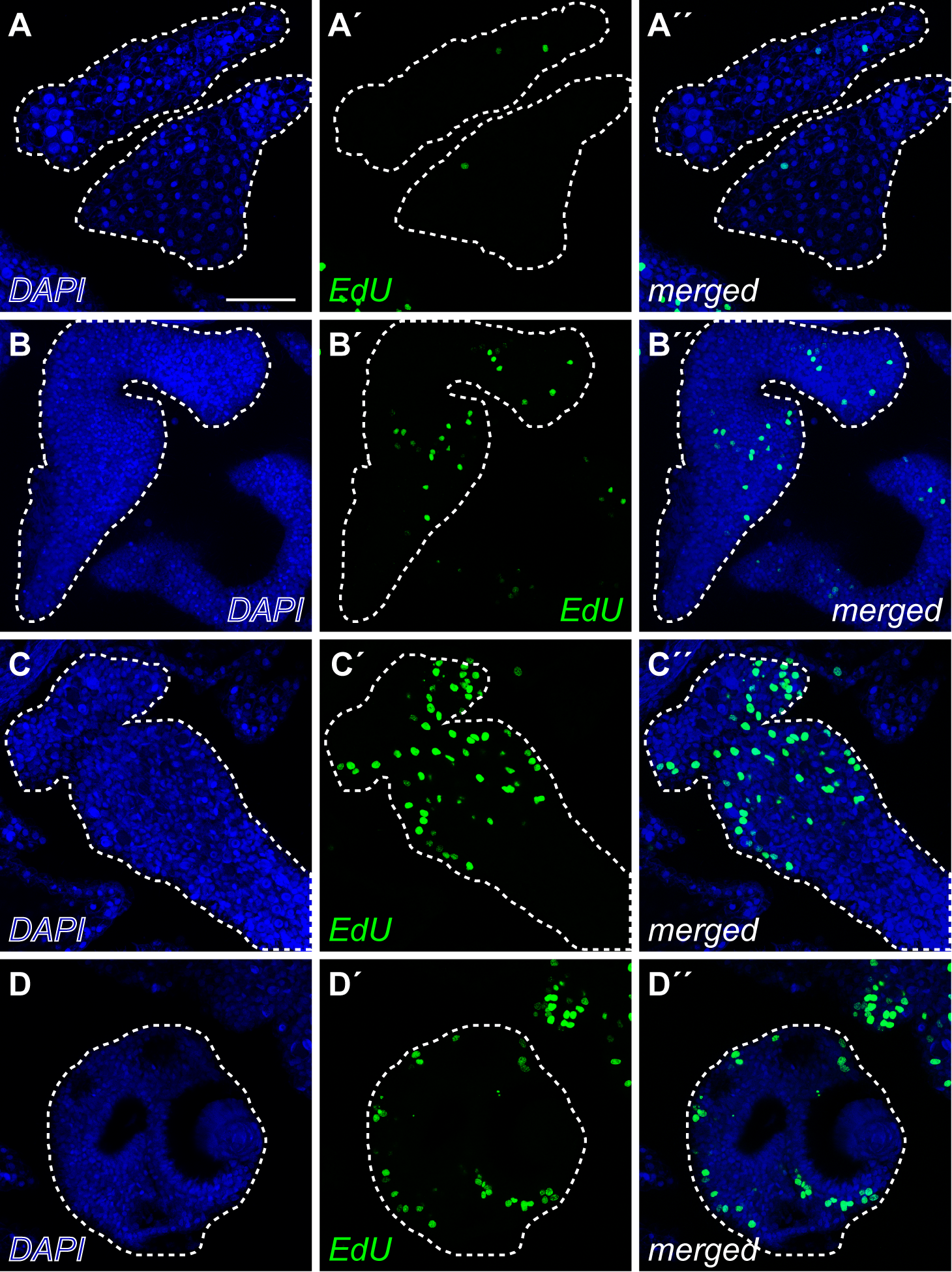

Supplement: Figure S2 — Micrographs used to calculate the percentage of S phase labeled cells. Four different body parts of juvenile medusae stained with DAPI (A–D) and EdU (A′-D′) in order to calculate the proliferation rates. 10 µm thick confocal stacks used for cell counts of DAPI- and EdU-stained cells in bell (A-A′′), manubrium (B-B′′), pedalium (C-C′′) and rhopalium (D-D′′). White dashed line indicates the area of cell counts. Scale bar, 50 µm (A) applies to all the pictures. (TIF) [file pone.0102628.s002.tif]

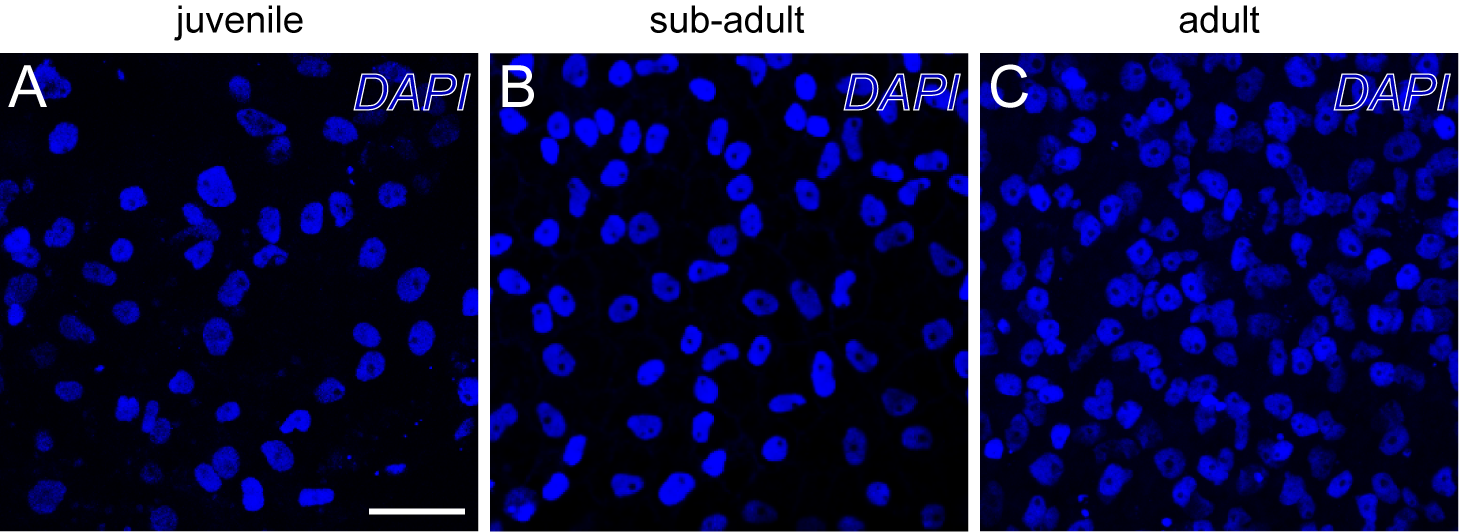

Supplement: Figure S3 — Cell density in the bell of Tripedalia cystophora changes with the age of the medusa. Bell of a juvenile, sub-adult and adult T. cystophora medusa stained with DAPI. The area of 100×100 µm used for nuclei counts in the bell of juvenile (A), sub-adult (B) and adult medusae (C). Scale bar, 20 µm (A) applies to all the pictures. (TIF) [file pone.0102628.s003.tif]
