# Supplementary material for: Cell Proliferation in Cubozoan Jellyfish Tripedalia cystophora and Alatina moseri
Source: PLoS One. 2014 Jul 21;9(7):e102628. doi: 10.1371/journal.pone.0102628 (PMC4105575; doi:10.1371/journal.pone.0102628)
Supplement: Table S1 — Counts of DAPI and EdU labeled cells in predefined areas of four different body parts of juvenile medusa of Tripedalia cystophora . Ratio of numbers of EdU to DAPI labeled cells results in the percentage of the S phase cells in the given body part (see also Figure S2). (DOCX) [file pone.0102628.s004.docx]

**Table S1.** Counts of DAPI and EdU labeled cells in predefined areas of four different body parts of juvenile medusa of *Tripedalia cystophora*. Ratio of numbers of EdU to DAPI labeled cells results in the percentage of the S phase cells in the given body part (see also Figure S2).

| body part | stain | # of cells | ratio EdU/DAPI |
| --- | --- | --- | --- |
| rhopalium 1 | DAPI | 727 | 12.6 % |
|  | EdU | 92 |  |
| rhopalium 2 | DAPI | 947 | 13.7 % |
|  | EdU | 130 |  |
| rhopalium 3 | DAPI | 559 | 13.0 % |
|  | EdU | 73 |  |
| rhopalium 4 | DAPI | 694 | 12.8 % |
|  | EdU | 89 |  |
| pedalium 1 | DAPI | 691 | 14.4 % |
|  | EdU | 100 |  |
| pedalium 2 | DAPI | 462 | 13.8 % |
|  | EdU | 64 |  |
| pedalium 3 | DAPI | 915 | 23.4 % |
|  | EdU | 215 |  |
| pedalium 4 | DAPI | 823 | 22.3 % |
|  | EdU | 184 |  |
| manubrium 1 | DAPI | 329 | 2.7 % |
|  | EdU | 9 |  |
| manubrium 2 | DAPI | 301 | 2.9 % |
|  | EdU | 9 |  |
| manubrium 3 | DAPI | 1182 | 4.7 % |
|  | EdU | 56 |  |
| manubrium 4 | DAPI | 816 | 3.9 % |
|  | EdU | 32 |  |
| bell 1 | DAPI | 299 | 1.0 % |
|  | EdU | 3 |  |
| bell 2 | DAPI | 330 | 1.8 % |
|  | EdU | 6 |  |
| bell 3 | DAPI | 443 | 2.2 % |
|  | EdU | 10 |  |
| bell 4 | DAPI | 349 | 2.5 % |
|  | EdU | 9 |  |
