# Supplementary material for: Cell Proliferation in Cubozoan Jellyfish Tripedalia cystophora and Alatina moseri
Source: PLoS One. 2014 Jul 21;9(7):e102628. doi: 10.1371/journal.pone.0102628 (PMC4105575; doi:10.1371/journal.pone.0102628)
Supplement: Table S2 — Counts of EdU labeled cells in predefined areas of 200×200 µm of four different body parts of mid-sized medusa Tripedalia cystophora during daytime and during nighttime (see also Figure 6 ). (DOCX) [file pone.0102628.s005.docx]

**Table S2.** Counts of EdU labeled cells in predefined areas of 200 x 200 µm of four different body parts of mid-sized medusa *Tripedalia cystophora* during daytime and during nighttime (see also Figure 6).

| body part | # of cells | |
| --- | --- | --- |
|  | daytime | nighttime |
| bell 1 | 11 | 3 |
| bell 2 | 10 | 42 |
| bell 3 | 9 | 33 |
| bell 4 | 13 | 12 |
| pedalium 1 | 24 | 63 |
| pedalium 2 | 14 | 41 |
| pedalium 3 | 8 | 43 |
| pedalium 4 | 7 | 32 |
| stalk base 1 | 4 | 35 |
| stalk base 2 | 29 | 95 |
| stalk base 3 | 11 | 88 |
| stalk base 4 | 10 | 82 |
| rhopalium 1 | 79 | 182 |
| rhopalium 2 | 82 | 220 |
| rhopalium 3 | 98 | 242 |
| rhopalium 4 | 116 | 367 |
